# Evaluation and Preparation of a Designed Kartogenin Drug Delivery System (DDS) of Hydrazone-Linkage-Based pH Responsive mPEG-Hz-*b*-PCL Nanomicelles for Treatment of Osteoarthritis

**DOI:** 10.3389/fbioe.2022.816664

**Published:** 2022-03-09

**Authors:** Wen-Ta Su, Ching-Cheng Huang, Hsia-Wei Liu

**Affiliations:** ^1^ Graduate Institute of Biochemical and Biomedical Engineering, National Taipei University of Technology, Taipei, Taiwan; ^2^ Department of Biomedical Engineering, Ming-Chuan University, Taipei, Taiwan; ^3^ Department Life Science, Fu Jen Catholic University, New Taipei City, Taiwan; ^4^ Graduate Institute of Applied Science and Engineering, Fu Jen Catholic University, New Taipei City, Taiwan

**Keywords:** kartogenin, hydrazone-linkage, methoxy poly(ethylene oxide), poly(ε-caprolactone), nanomicelles

## Abstract

Osteoarthritis (OA) is a chronic disease caused by the damage of articular cartilage. Kartogenin (KGN) is a well-recognized small molecule which could induce MSCs chondrogenesis and promote cartilage repair treatments. Nano-level micells could be a suitable drug carrier technology for the treatments. In this study, the acid-responsive methoxy poly(ethylene oxide)-hydrazone-poly(ε-caprolactone) copolymers, mPEG-Hz-*b*-PCL, were synthesized. The structure was characterized by ^1^H NMR. The evaluation of a designed kartogenin drug delivery system (DDS) of hydrazone-linkage-based pH responsive mPEG-Hz-*b*-PCL nanomicelles for treatment of osteoarthritis could be carried out.

## Introduction

Osteoarthritis (OA) is a common disease, which is characterized by articular cartilage destruction and local inflammation, resulting in pain and disability (Hunziker et al., 2002). Kartogenin (KGN) is a well-recognized small molecule which could induce MSCs chondrogenesis and improve damaged cartilage repair without the disadvantages of growth factors (Kang et al., 2014; Li et al., 2016a; Li et al., 2016b; Shi et al., 2016; Hu et al., 2017; Fan et al., 2018; Im, 2018; Cai et al., 2019; Xu et al., 2019; Yang et al., 2019; Zheng et al., 2019; Silva et al., 2020). The loading efficiency and release kinetics of the biomolecules depended on the initial concentrations of the nanospheres, as well as the hydrophobicity of the loaded molecules, resulting in controllable and programmed delivery profiles.

Recently, several designed biomaterial systems had been developed to promote the sustained release of KGN molecule towards cartilage and osteochondral regeneration (Kang et al., 2014; Li et al., 2016a; Li et al., 2016b; Shi et al., 2016; Hu et al., 2017; Fan et al., 2018; Im, 2018; Cai et al., 2019; Xu et al., 2019; Yang et al., 2019; Zheng et al., 2019; Silva et al., 2020). For these reasons, numerous materials had been proposed, modified and employed for the medical applications such as synthetic materials and natural materials (Vogel and Trotter, 1987; Liaw et al., 1999; Baldwin and Kiick, 2010; Valencia et al., 2018; Huang, 2021; Shin et al., 2021). Also, polycaprolactone-containing materials were used to promote a sustained release of KGN molecule as a polymeric drug delivery system (Kang et al., 2014; Li et al., 2016b; Shi et al., 2016; Hu et al., 2017; Fan et al., 2018; Im, 2018; Cai et al., 2019; Yang et al., 2019; Zheng et al., 2019).

Stimuli-responsive polymeric drug delivery systems using various triggers to release the KGN molecule at the sites would be an important area (Vogel and Trotter, 1987; Liaw et al., 1999; Baldwin and Kiick, 2010; Kang et al., 2014; Li et al., 2016b; Shi et al., 2016; Hu et al., 2017; Fan et al., 2018; Im, 2018; Valencia et al., 2018; Cai et al., 2019; Yang et al., 2019; Zheng et al., 2019; Das et al., 2020; Silva et al., 2020; Huang, 2021; Shin et al., 2021). Among various stimuli-responsive materials, designed pH-responsive materials had been studied extensively (Na et al., 2004; Bae et al., 2005; Gillies and Frechet, 2005; Bae et al., 2005; Kim et al., 2005; Gao et al., 2005; Lee et al., 2005a; Lee et al., 2005b; Sawant et al., 2006; Lee et al., 2007; Chen et al., 2012; Qi et al., 2017; Qi et al., 2018). Designed pH-responsive materials would be employed as pH-responsive drug delivery systems. A specific chemical functional group designed and introduced in the chemical structure could be sensitive and be responsive to pH changes of the drug-releasing environments (Lee et al., 2007; Chen et al., 2012; Qi et al., 2017; Qi et al., 2018). Further, the specific chemical functional group of a hydrazone linkage could be significant synthons for numerous transformations and have gained importance in pharmaceutical and medical sciences due to their various clinical applications. Hence, the hydrazone linkages could be considered and employed in the drug delivery vehicles for pH-responsive drug delivery system (Zhou et al., 2011; Chen et al., 2012; Zhou et al., 2012; Koutroumanis et al., 2013; Zhang et al., 2013; Qi et al., 2017; Qi et al., 2018).

In this study, the synthesis and characterization of diblock polymeric materials with a hydrazine linkage were carried out and applied in pH-responsive drug delivery systems. A pH-responsive cleavable methoxy polyethylene glycol-hydrazone-block-polycaprolactone (mPEG-Hz-*b*-PCL) copolymer would be designed as a modified pH-responsive drug carrier that would selectively decompose under acidic conditions. The amphiphilic mPEG-Hz-*b*-PCL could establish a polymeric nano-micelle for loading KGN, which would be applied for OA treatments. Further, the physicochemical properties of the pH-responsive drug carrier would be characterized and the release behavior and cytotoxicity of the resulting KGN-loaded nano-micelles would be investigated.

## Materials and Methods

### Materials

The chemicals used in the work, such as 2-[(Biphenyl-4-yl)carbamoyl]benzoic acid(Kartogenin, KGN) (Sigma-Aldrich Company, United Kingdom), 1-Dodecanol (Alfa Aesarm, United States), ε-caprolactone (Alfa Aesarm, United States), methoxyl PEG hydrazide 5k (NANOCS, United States), petrol (Sigma-Aldrich Company, United Kingdom), stannous octanoate (Sigma-Aldrich Company, United Kingdom), acetonitrile (ACN) (Sigma-Aldrich Company, United Kingdom), ether (J.T.Baker, United States), pyrene (Sigma-Aldrich Company, United Kingdom), dichloromethane (Mallinckrodt, United States), Dess-Martin periodinane (solution in 0.3M dichloromethane) (Sigma-Aldrich Company, United Kingdom), dimethyl sulfoxide (Sigma-Aldrich Company, United Kingdom), and ethanol (Merck, Germany).

### Preparation of Hydroxy-Terminated Poly(ε-Caprolactone) (PCL-OH)

**FIGURE 1:**
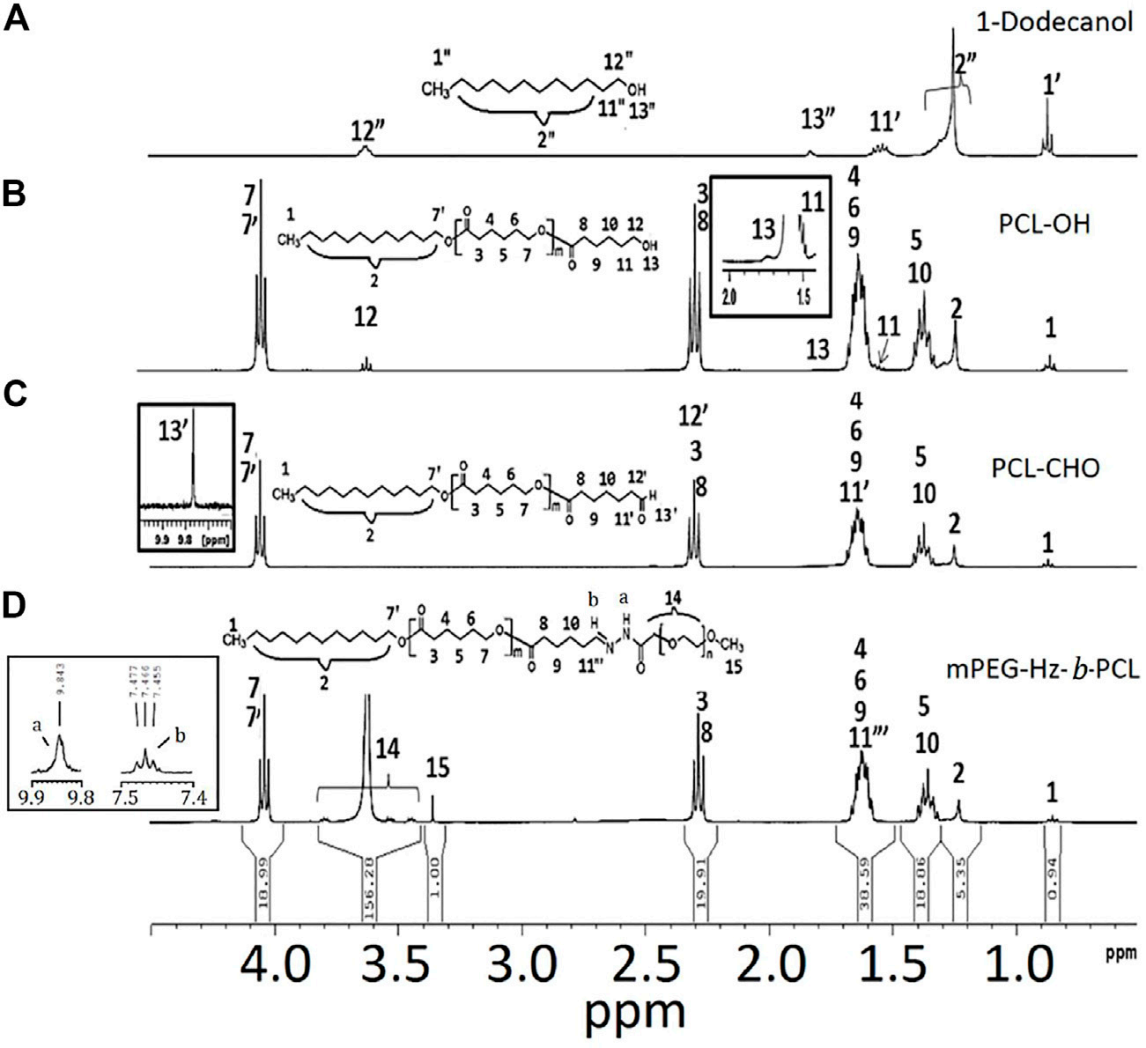
^1^H NMR spectrum of the samples: **(A)** 1-dodecanol, **(B)** PCL-OH, **(C)** PCL-CHO, and **(D)** mPEG-Hz-*b*-PCL.

The PCL-OH could be synthesized by using the ring-opening polymerization (ROP) of ε-caprolactone (CL) and 1-dodecanol using stannous octanoate [Sn(Oct)_2_] as the catalyst (Scheme 2). In ROP of CL, 1-dodecanol (1.79 × 10^–3^ mol) and CL (0.18 mol) was kept in a 100 ml Schlenk tube equipped with a magnetic bar under dry N_2_ atmosphere. Then, 5.24 × 10^–2^ mmol of Sn(Oct)_2_ was added. The reaction mixture was further degassed via three freeze-pump-thaw cycles and then immersed in an oil bath at 110 °C for 24 h. The ROP could be stopped by freezing the reaction mixture with liquid N_2_. Further, the resulting product was dissolved in a dichloromethane and precipitated from petroleum ether. The precipitated PCL-OH could be collected by centrifugation, purified by repeated dissolution in dichloromethane, precipitated from petroleum ether twice, and dried under vacuum at r.t. Yield = 96%. ^1^H NMR (300 MHz, CDCl_3_) (Brukwe Avance-300 MHz) (Figure 1): δ (ppm) = 1.25(H_2_), 1.31–1.45 (H_5_, H_10_), 1.51–1.68(H_4_,H_6_,H_9_,H_11_), 1.74(H_13_), 2.31(H_3_,H_8_), 3.62 (H_12_), 4.05(H_7_,H_7’_).

### Preparation of PCL-CHO

Dess–Martin periodinane (DMP oxidation) is a selective method for oxidizing primary alcohols to aldehydes. Oxidation of PCL-OH to the corresponding aldehyde-terminated PCL-CHO by DMP have been carried out for 6 h. The crude product is dissolved in a 20 ml dichloromethane and precipitated from 400 ml petroleum ether. The precipitated PCL-OH is collected by centrifugation. The separated PCL-CHO is purified by repeated dissolution in dichloromethane, precipitated from petroleum ether twice, and finally is dried under vacuum at room temperature for 24 h. Yield = 91%. ^1^H NMR (300 MHz, CDCl_3_) (Figure 1C); δ (ppm) = 1.25(H_2_), 1.31–1.45 (H_5_, H_10_), 1.51–1.68(H_4_,H_6_,H_9_,H_11’_), 1.74(H_13_), 2.31(H_3_,H_8_,H_12’_), 3.62 (H_12_), 4.05(H_7_,H_7’_),9.78 (H_13’_).

### Preparation of mPEG-Hz-*b*-PCL

**SCHEME 1:**
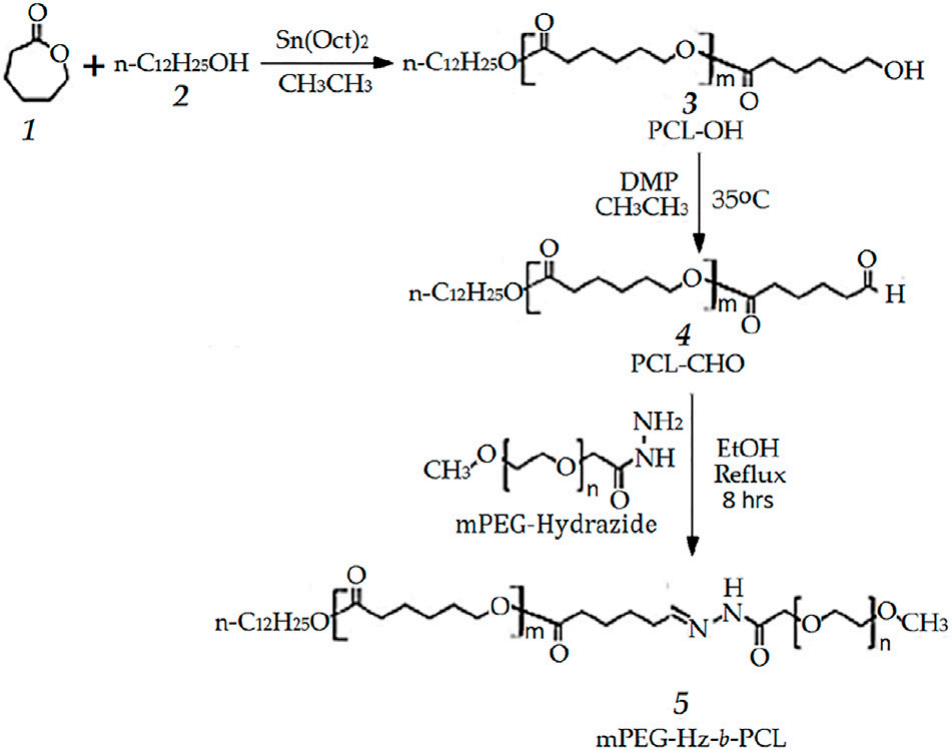
Preparation of methoxy polyethylene glycol-hydrazone-block-polycaprolactone block copolymer.

Alcohol solutions of 298 mg PCL-CHO and 400 mg mPEG-hydrazide were mixed together at 37°C. The formation of hydrazone linkages was carried out by refluxing for 8 h(Scheme 1). The resulting mPEG-Hz-*b*-PCL was precipitated from 400 ml petroleum ether. ^1^H NMR (300 MHz, CDCl_3_) (Figure 1C); δ (ppm) = 1.25(H_2_), 1.31–1.45 (H_5_, H_10_), 1.51–1.68(H_4_,H_6_,H_9_,H_11″_), 1.74(H_13_), 2.31(H_3_,H_8_),3.38 (H_15_), 3.42–3.82 (H_14_), 4.05(H_7_,H_7′_).

### Critical Micelle Concentration of mPEG-Hz-*b*-PCL

Critical micelle concentration (CMC) value of the resulting mPEG-Hz-*b*-PCL polymer could be determined by a pyrene fluorescent probe method. The mPEG-Hz-*b*-PCL aqueous solutions with various concentrations of 2.5 × 10^–1^, 1.25 × 10^–1^, 7.50 × 10^–2^, 5 0.00 × 10^–2^, 1.25 × 10^–2^, 6.25 × 10^–3^, 3.13 × 10^–3^, 1.56 × 10^–3^, 1.00 × 10^–3^, and 5.00 × 10^–4^ mg/ml were prepared. All the solutions were sonicated and equilibrated in a water bath for 1 h. The samples were then kept at room temperature for 24 h and the emission spectra of the mPEG-Hz-b-PCL aqueous solutions were measured from 290 to 350 nm by using a fluorescence spectrophotometer (SCINCO Fluorescence Spectrophotometer). The ratios of the peak intensities of the excitation spectra of pyrene at 337 nm (I_337_) and 334 nm (I_334_) were further analyzed. The I_337_/I_334_ ratios were obtained as a function of the concentrations. An interception point of the two tangent straight lines was observed at a low concentration as a CMC value.

### *In vitro* Cytotoxicity of Drug-free mPEG-Hz-*b*-PCL Nanomicelles

For the cell viability test, the original medium was refreshed and free nanomicelle of predetermined concentrations were added and incubated at 37°C under 5% CO_2_ for 24 h. Human SW1353 cells were used as assay cells. All cell lines were seeded into a 96-well plate at a density of 5 × 10^3^ cells/well in complete DMEM. The medium in each well was replaced with a fresh culture cell medium, and MTT [3-(4,5-dimethyl-2-thiazolyl)-2,5-diphenyl-2H-tetrazolium bromide] reagent (5 mg/ml) was added to each well and incubated for a further 4 h. Finally, DMSO (100 µl) was added to each well and the plates were incubated at 37°C for 25 min. Cell viability was measured according to the following formula: (live cells/total cells) × 100% (*n* = 5). Similarly, the cytotoxicity of the blank mPEG-Hz-b-PCL copolymer against SW1353 cell lines was assessed at a concentration of 6.25 × 10^−3^ mg/ml(CMC of mPEG-Hz-*b*-PCL), 6.25 × 10^−2^ mg/ml (10 × CMC of mPEG-Hz-*b*-PCL), and 6.25 × 10^−1^ mg/ml (100 × CMC of mPEG-Hz-*b*-PCL)µg/ml by following the aforementioned protocol.

### Preparation of KGN-Loaded mPEG-Hz-*b*-PCL Nanomicelles by Using Solvent Evaporation Method

Preparation of KGN-loaded mPEG-Hz-*b*-PCL nanomicelles solution could be carried out by dissolution of 10 mg of KGN molecule and 50 mg of amphiphilic mPEG-Hz-*b*-PCL block copolymer in 5 ml acetone to prepare a mixed solution. The mixed solution was slowly added to 200 ml distilled water under vigorous stirring to induce formation of nanomicelle solution. The nanomicelle solution was open to air and stirred overnight to remove acetone. Further, the nanomicelle solution was diluted with H_2_O to prepare desired concentrations in a dark environment to afford KGN-loaded mPEG-Hz-*b*-PCL nanomicelles (NMs). The amount of KGN loaded in the cores of nanomicelles could be determined based on a calibration curve of free KGN. The drug loading efficiency (DLE) and drug loading capacity (DLC) of mPEG-Hz-*b*-PCL micelles were calculated using the following equations (1) and (2):

Equation (1): DLC% = the weight of KGN in the nanomicelles/the weight of KGN-loaded nanomicelles ×100%
Equation (2): DLE% = the weight of KGN in the nanomicelles/the weight of KGN fed initially × 100%

The size and size distribution of blank polymeric micelles, mPEG-Hz-*b*-PCL block copolymer nanomicelles, and KGN-loaded mPEG-Hz-*b*-PCL block copolymer nanomicelles were measured using the dynamic light scattering (DLS) (Malvern Zetasizer Nano ZS90).

### KGN Molecule Released From the KGN-Loaded mPEG-Hz-*b*-PCL Nanomicelle

The *in vitro* release of KGN molecule from different hydrazone-linkage based mPEG-Hz-*b*-PCL block copolymer solutions was determined using HPLC. The release medium was 45 ml of 0.1 M phosphate buffer (pH 5.0 and 7.4), providing sink conditions for KGN molecule, and maintained at 37°C. At various time intervals, 2 ml of PBS solution of KGN-loaded mPEG-Hz-*b*-PCL nanomicelle was collected and replaced by fresh PBS with the same amount. The total amount of released KGN molecule from the KGN-loaded hydrazone-linkage based mPEG-Hz-*b*-PCL nanomicelle could be determined by HPLC. The amount of KGN molecule released from the KGN-loaded nanomicelle was calculated at specific time intervals.

## Results

### ^1^H NMR Analysis of Methoxy Polyethylene Glycol-Hydrazone-Block-Polycaprolactone Block Copolymer

In this study, methoxy polyethylene glycol-hydrazone-*block-*polycaprolactone(mPEG-Hz-*b*-PCL) block copolymer could be prepared as shown in Scheme 1. At first, PCL-OH polymer was synthesized via ROP of ε-CL in bulk at 110 °C using 1-dodecanol as the initiator and Sn(Oct)_2_ as the catalyst. ^1^H-NMR spectrum(CDCl_3_) could be employed to determine the content of polycaprolactone(PCL) segment, chemical structure, and number-averaged molecular weight(*M*_n_) of the resulting PCL-OH polymer. From observation of Figure 1B, the characteristic peaks at 1.39 ppm, 1.65 ppm, 2.29–2.33 ppm, and 4.06 ppm, the characteristic peak at 1.25 ppm, the characteristic peak at 1.72 ppm, and the characteristic peak at 1.55 ppm were assigned to -CH_2_- protons in the PCL segment, -CH_2_- protons in the dodecanyl segment, -OH proton(H_13_), and -CH_2_-CH_2_-OH protons(H_11_) of the resulting PCL-OH polymer, respectively. Further, the number-averaged molecular weight (*M*_n_) of the PCL-OH could be determined from the corresponding ^1^H NMR spectrum(CDCl_3_) by comparing the integrated peak area of aliphatic methylene protons of the backbone PCL chain(H_3_ and H_8_) at ∼2.3 ppm [(2m + 2)H] to that of methyl proton(H_11′_) in dodecanyl group at 0.8 ppm (3H) and methylene proton(H_12_) near hydroxyl end group end at 3.65 ppm(2H) (Figure 1B). The number-averaged molecular weight (*M*_n_) of the PCL-OH polymer could be calculated by using the ratio between the characteristic peak areas at 2.3 and 0.8 ppm and the ratio between the peak areas at 2.3 ppm and 3.65 ppm. The number-averaged molecular weight (*M*_n_) and “m” value of PCL-OH copolymer could be determined to be 2,496 and 19, respectively. Typical ^1^H NMR spectrum of PCL-OH polymer in CDCl_3_ solvent (Figure 1B) clearly showed the presence of characteristic peaks corresponding to the methylene groups of backbone PCL segments and dodecanyl group of the initiator(1-dodecanol). The resulting PCL-OH polymer are oxidized to be PCL-CHO polymer and then reacted with mPEG5000-hydrazide (Scheme 1). Typical ^1^ H NMR spectrum of the PCL-CHO polymer was shown in Figure 1C. The conversion of hydroxyl end group into the corresponding aldehyde end group is confirmed by the appearance of the new characteristic peak attributed to the terminated proton (H_13′_) of aldehyde end group in PCL-CHO polymer at 9.78 ppm and disappearance of terminated proton(H_12_) of hydroxyl end group in PCL-OH polymer at 3.68 ppm after oxidation. Almost quantitative conversion of–OH end group into its corresponding aldehyde end group was observed by comparing Figures 1B,C. After reaction of PCL-CHO polymer with mPEG-hydrazide, a new hydrazone-linkage based pH responsive mPEG-Hz-*b*-PCL block copolymer could be obtained. The ^1^H NMR spectrum of the synthesized mPEG-Hz-*b*-PCL block copolymer was showed in Figure 1D. The formation of mPEG-Hz-*b*-PCLblock co polymer could be confirmed from the disappearance of terminated proton (H_13′_) of aldehyde end group in PCL-CHO polymer at 9.78 ppm and appearance of terminated proton(H_15_) of methoxyl end group in mPEG-Hz-*b*-PCL block copolymer at 3.36 ppm*.* The characteristic peaks at 1.37, 1.64, 2.30, and 4.05 ppm were assigned to the methylene protons of -O(O)CCH_2_CH_2_CH_2_CH_2_CH_2_O(H_4_,H_6_,H_9_), -CH_2_CH_2_CH_2_CH_2_CH_2_(H_5_,H_10_), -OCCH_2_(H_3_,H_8_), and -CH_2_O(O)C(H_7_,H_7′_) in the PCL segment of mPEG-Hz-*b*-PCL block polymer. The characteristic peaks at 3.38 and 3.65 ppm were attributed to the signals of CH_3_O- and -CH_2_CHO- in the mPEG segment of mPEG-Hz-*b*-PCL block copolymer. The number average molecular weight (Mn) of the mPEG-Hz-*b*-PCL block copolymer could be calculated from ^1^H NMR spectrum as shown in Figure 1D. Also, the methoxy polyethylene glycol (mPEG) weight fraction of the mPEG-Hz-*b*-PCL block copolymer could be calculated from the results of ^1^H NMR spectrum. The area ratio of PCL segment/mPEG segment was calculated to be a ratio of 33%/67% ˜ 19/38 by calculation of area of the characteristic peak at 4.0 ppm (PCL segment) and the characteristic peak at 3.58 ppm (mPEG segment). The “m” value of copolymer had been determined to be 19 as described above. The formation of the mPEG_38_-Hz-*b*-PCL_19_ structure would be estimated.

### Self-Assembly and Critical Micelle Concentration of Hydrazone-Linkage Based pH Responsive mPEG-Hz-*b*-PCL Block Polymer

**FIGURE 2:**
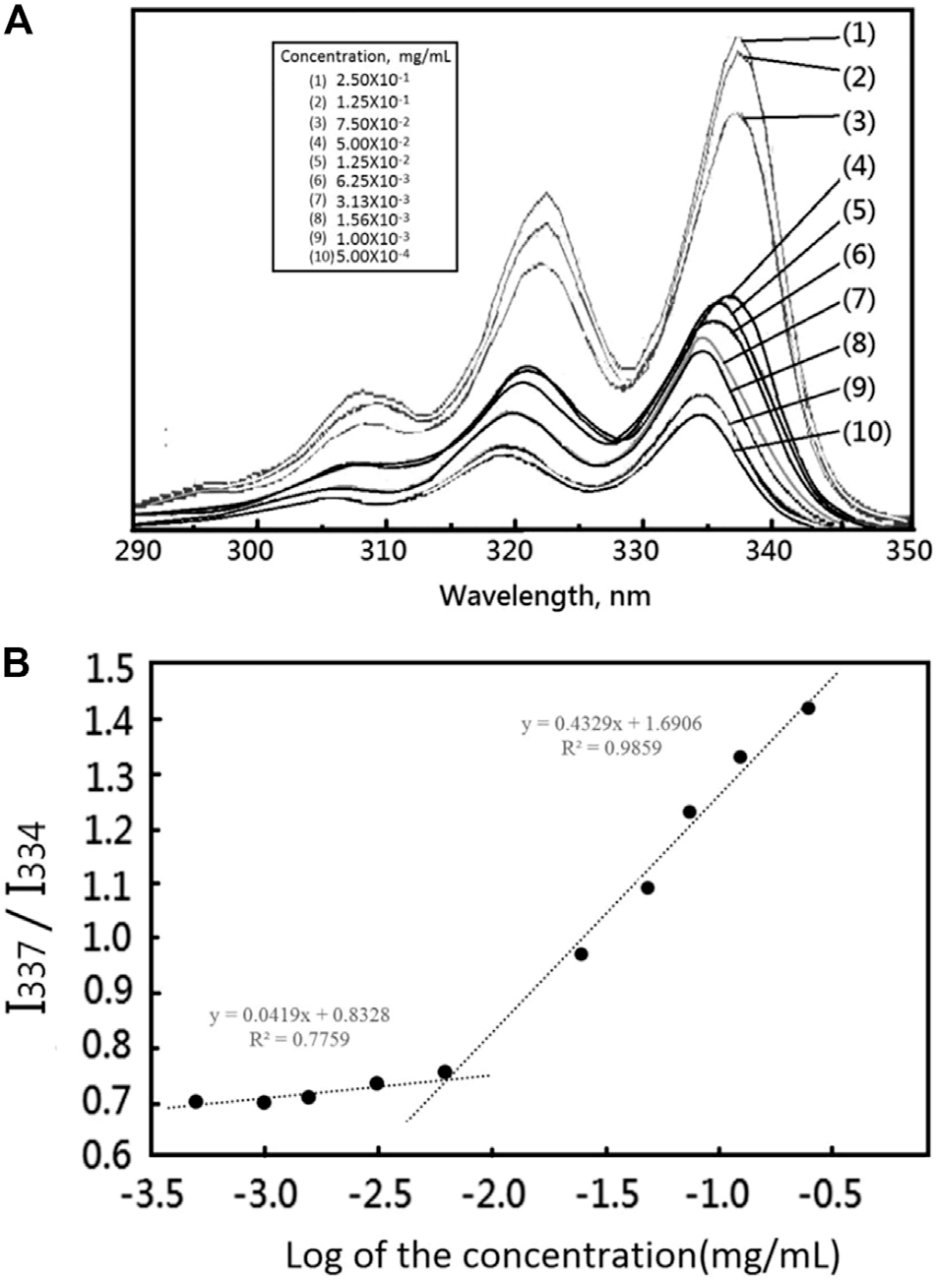
**(A)** The fluorescence emission spectra of pyrene as a function of copolymer concentration in distilled water (mg/mL) ([Pyrene] = 6.0 × 10^−7^ M, λex = 390 nm). **(B)** The fluorescence intensity ratio of I_337_/I_334_ from pyrene emission spectra vs. the log of the concentration (logC:mg/mL), a measurement used to determine the CMC for mPEG-Hz-*b*-PCL.

Importantly, the stability of polymeric nanomicelles at extreme dilution, such as the bloodstream, is imperative for their clinical applications. Critical micelle concentration (CMC) could be employed to study the solution properties of the designed amphiphilic mPEG-Hz-*b*-PCL block copolymers. In this study, the CMC could be estimated based on the fluorescent intensity of hydrophobic pyrene as a fluorescent probe embedded in the hydrophobic core of mPEG-Hz-*b*-PCL block copolymeric nanomicelles by using steady-state fluorescence measurement (Liaw et al., 1999). The emission band intensity of pyrene probe was low at relative low polymer concentrations which clearly indicated that pyrene was predominantly located in a relatively polar aqueous environment since the pyrene probes were not encapsulated in the nonpolar hydrophobic cores(PCL segments) of mPEG-Hz-*b*-PCL block copolymeric nanomicelles (Figure 2A). Above CMC, nanomicelles of amphiphilic mPEG-Hz-*b*-PCL block copolymers could form spontaneously because of a thermodynamic balance between enthalpy and entropy (Liaw et al., 1999). The emission band intensity rapidly increased with an increase in the concentration of the amphiphilic mPEG-Hz-*b*-PCL block copolymer above the CMC as pyrene was entrapped in the nonpolar cores (PCL segments) of a growing number of self-assembled nanomicelles as shown in Figure 2A. Hence, CMC could be observed from the plot of emission band intensity ratios (I_337_/I_334_) of pyrene probes against polymer concentration of amphiphilic mPEG-Hz-*b*-PCL aqueous solutions (Figure 2B). The CMC of mPEG-Hz-*b*-PCL aqueous solution was found to be a relative low value of 6.25 × 10^−3^ mg/ml at room temperature which would indicate the formation of stable polymeric nanomicelles in aqueous environment at a low copolymer concentration as a result of the strong hydrophobicity of amphiphilic mPEG-Hz-*b*-PCL block copolymers. The linearity was almost maintained between 5.00 × 10^−4^ mg/ml and 6.25 × 10^−3^ mg/ml of mPEG-Hz-*b*-PCL concentration (Figure 2B). Above 6.25 × 10^−3^ mg/ml, a sharp increase of emission band intensity ratio (I_337_/I_334_) was observed. The CMC was a concentration of amphiphilic mPEG-Hz-*b*-PCL block copolymer above which the mPEG-Hz-*b*-PCL unimers started to self-assemble to form a liquid colloidal solution. The CMC would be attributed to the beginning of aggregation of the polymeric chains, resulting nanomicelles of amphiphilic mPEG-Hz-*b*-PCL block copolymer. When amphiphilic mPEG-Hz-*b*-PCL solutions were diluted at concentrations below CMC, the liquid colloidal solution would dissociate into mPEG-Hz-*b*-PCL unimers.

### *In vitro* Cytotoxicity of Drug-Free mPEG-Hz-*b*-PCL Nanomicelles

**FIGURE 3:**
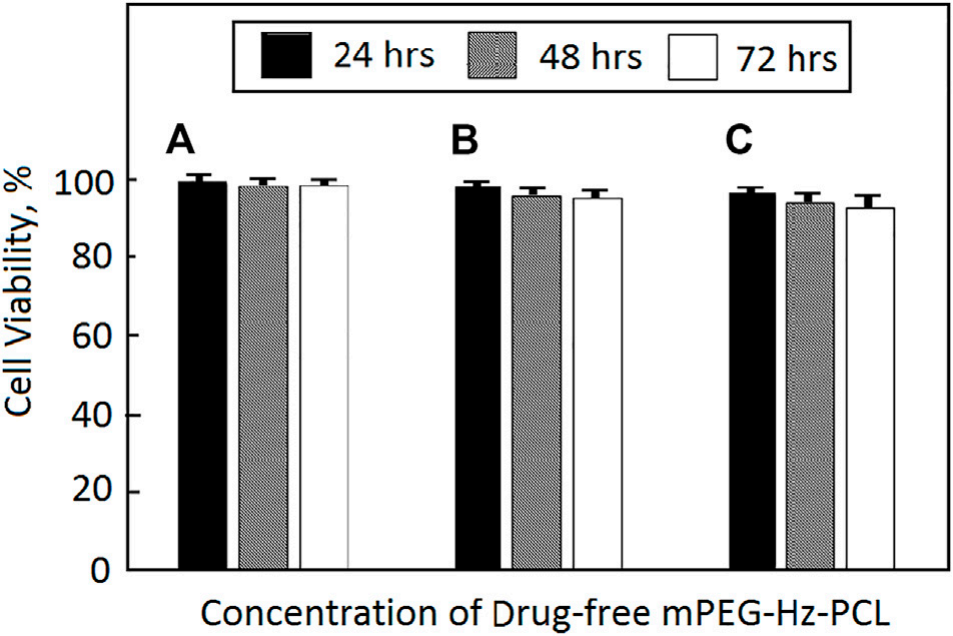
Cytotoxicity of SW1353 cells against drug-free mPEG-Hz-*b*-PCL nano-micelle determined by MTT assay (*p < 0.05) at different concentrations: **(A)** CMC (6.25 × 10^−3^ mg/ml), **(B)** 10 × CMC (6.25 × 10^−2^ mg/ml), and **(C)** 100 × CMC (6.25 × 10^−1^ mg/ml).

SW1353 cells were used to evaluate the cytotoxicity of drug-free mPEG-Hz-*b*-PCL nanomicelles. After 24, 48, and 72 h incubation with drug-free mPEG-Hz-*b*-PCL nanomicelles, the results of cell viabilities could be observed as shown in Figure 3. The drug-free mPEG-Hz-*b*-PCL nanomicelles were biocompatible and showed a slight decrease in cellular viability of SW1353 cells with an increasing concentration of the mPEG-Hz-*b*-PCL nanomicelle. The cell viability of SW1353 decreased from ca.99.5% for mPEG-Hz-*b*-PCL nanomicelles at CMC (6.25 × 10^−3^ mg/ml) and ca.99.0% for mPEG-Hz-*b*-PCL nanomicelles at 10 × CMC(6.25 × 10^−2^ mg/ml), to ca.98.1% for mPEG-Hz-*b*-PCL nanomicelles at 100 × CMC(6.25 × 10^−1^ mg/ml) after 24 h of incubation. Similarly, the cell viability of SW1353 decreased from ca.99.1% for mPEG-Hz-b-PCL nanomicelles at CMC, ca.97.8% for mPEG-Hz-*b*-PCL nanomicelles at 10 × CMC, to ca.96.7% for mPEG-Hz-*b*-PCL nanomicelles at 100 × CMC after 48 h of incubation and from ca.99.1% for mPEG-Hz-*b*-PCL nanomicelles at CMC, ca. 97.1% for mPEG-Hz-*b*-PCL nanomicelles at 10 × CMC, to ca.96.1% for mPEG-Hz-*b*-PCL nanomicelles at 100 × CMC after 72 h of incubation. These results indicated that relative low cytotoxicity of drug-free new designed mPEG-Hz-*b*-PCL nanomicelles for DDS was obtained. Kartogenin (KGN) was a non-toxic and stable small molecule (Johnson et al., 2012). Hence, drug-free mPEG-Hz-*b*-PCL nanomicelles with low cytotoxicity could be expected to be employed to form KGN-loaded mPEG-Hz-*b*-PCL nanomicelles with low cytotoxicity.

### Size and Size Distribution of mPEG-Hz-*b*-PCL

Amphiphilic copolymers would aggregate and form polymeric nanomicelles in aqueous solutions as a drug delivery system (DDS). Size and size distribution of polymeric nanomicelles would be considered as an important factor governing the pharmacodynamics properties of drug-loaded polymeric nanomicelles in clinic applications. In this study, a new amphiphilic mPEG-Hz-*b*-PCL copolymer was designed and synthesized. At a higher concentration than CMC of amphiphilic mPEG-Hz-*b*-PCL copolymer solution, the amphiphilic mPEG-Hz-*b*-PCL copolymer would introduce a self-assembling behavior into nanomicellar aggregates. The designed amphiphilic mPEG-Hz-*b*-PCL copolymer containing hydrophilic mPEG segments and hydrophobic PCL segments would enhance the self-assembling behaviors for formation of polymeric nanomicelles in an aqueous solution.

Hydrodynamic diameters and size distributions (PDI) of the blank polymeric nanomicelles of mPEG-Hz-*b*-PCL (5.00 × 10^−1^ mg/ml), which was lower than 100×CMC (6.25 × 10^−1^ mg/ml), were determined by DLS after various time intervals. After 24 h, the hydrodynamic diameters were kept in a range from 99 to 101 nm. The diameter has almost not changed, considering the accuracy of the measurements. Polymeric nanomicelles of mPEG-Hz-*b*-PCL would be maintained a constant PDI value of 0.16 during 24 h indicating that mPEG-Hz-*b*-PCL could establish a stable polymeric nanomicelle in aqueous solution as a drug carrier.

**SCHEME 2:**
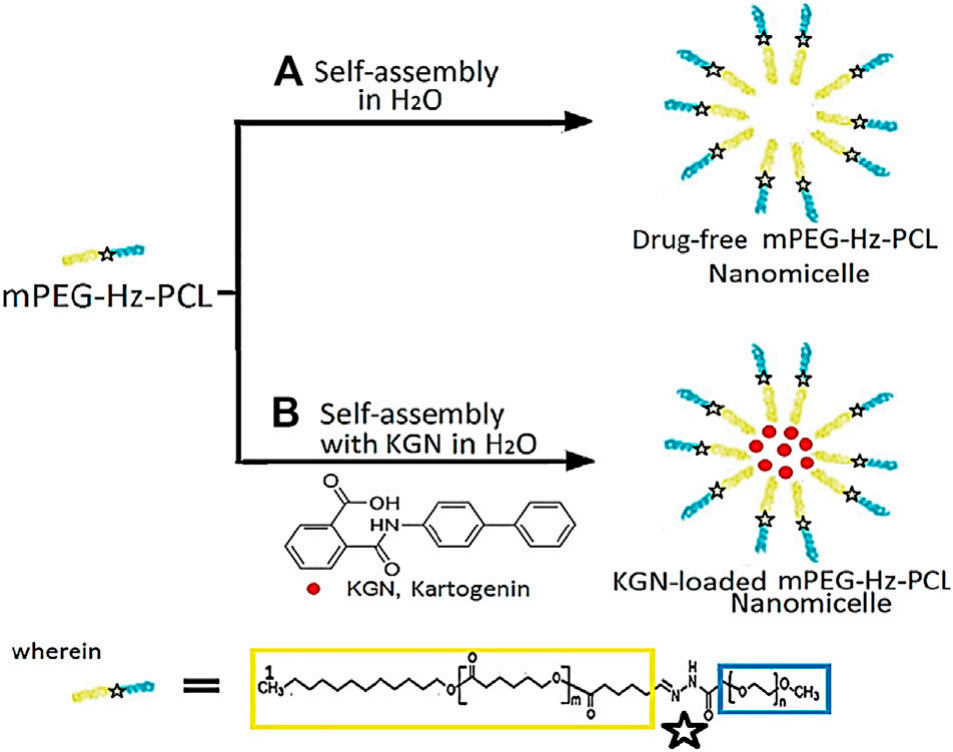
Molecular structure of (mPEG-Hz-*b*-PCL) block copolymers and KGN-loaded mPEG-Hz-*b*-PCL micelles.

During the solvent exchange process, diffusion of acetone in an aqueous medium forces the mPEG-Hz-*b*-PCL block to undergo microphase separation and led to the formation of polymeric nanomicelles. Molecular structures of mPEG-Hz-*b*-PCL block copolymers and KGN-loaded mPEG-Hz-*b*-PCL nanomicelles were illustrated in Scheme 2. The size and size distribution of blank polymeric nanomicelles, mPEG-Hz-*b*-PCL block copolymer nanomicelles, and KGN-loaded mPEG-Hz-*b*-PCL block copolymer nanomicelles were measured using the dynamic light scattering (DLS) as shown in Table 1. According to the DLS results, monodispersed mPEG-Hz-*b*-PCL polymeric nanomicelles (PDI = 0.16 ± 0.02) were obtained. A slight increase in the hydrodynamic diameter of nanomicelles was recorded when KGN molecules were loaded in the hydrophobic cores of polymeric nanomicelles. When the strong hydrophobic drug was loaded in the polymeric nanomicelles and strong hydrophobic drug-loaded polymeric nanomicelles were formed. Strong hydrophobic drug-loaded polymeric nanomicelles showed higher hydrodynamic diameter value (168 nm) as compared to the drug-free polymeric nanomicelles (116 nm). The obvious hydrodynamic diameter difference observed between strong hydrophobic drug-loaded polymeric nanomicelles and drug-free polymeric nanomicelles might be attributed to the strong hydrophobic association, resulting in a compacted microstructure of nanomicelle with a relative small hydrodynamic diameter. KGN molecule was loaded by mPEG-Hz-*b*-PCL polymeric nanomicelles to improve the drug’s poor water solubility. The small polydispersity index(PDI) of 0.16 ± 0.02 and 0.23 ± 0.03 indicated uniform diameter distributions of the polymeric nanomicelles with/without KGN molecule(Table 1). In usual, copolymer micelles containing mPEG-PCL segments were spherical in shape which could be determined by using transmission electron microscopy (TEM) (Qi et al., 2017; Zhu et al., 2017; Qi et al., 2018; Zhang et al., 2019; Wang et al., 2020; Park et al.,2021). Wang et al. reported that transmission electron microscopy (TEM) images of drug-loaded pH-controlled delivery micelles containing mPEG-PCL polymeric segments could be exhibited spherical in shape (Zhu et al., 2017). Zhang et al. reported that the micelle morphologies of copolymer micelles containing mPEG-PCL segments were investigated by TEM and the images clearly indicated that the diselenide or disulfide mPEG-PCL micelles have a spherical shape which were in good agreement with DLS results of these micelles (Wang et al., 2020). Also, Park et al. reported that TEM images of Cyclic RGDfK- and Sulfo-Cy5.5-functionalized mPEG-PCL theranostic nanosystems containing mPEG-PCL segments showed a spherical shape (Zhang et al., 2019).

**TABLE 1:** Sizes and size distributions of mPEG-Hz-*b*-PCL nanomicelles and KGN-loaded mPEG-Hz-*b*-PCL nanomicelles.

Sample	Size diameter ±SD(nm) (*n* = 3)	Size distribution(PDI) ± SD (*n* = 3)
mPEG-Hz-*b*-PCL nanomicelles	138 ± 13	0.16 ± 0.02
KGN-loaded mPEG-Hz-*b*-PCL nanomicelles	168 ± 11	0.23 ± 0.03

### KGN Drug Loading Efficiency

In the aim to investigate the specific release characteristics of the prepared nanomicelles, the cumulative drug release from mPEG-Hz-b-PCL nanomicelles was determined. The KGN drug loading (DL) of KGN molecule was detected by HPLC. The percentage of KGN loaded in the mPEG-Hz-*b*-PCL nanomicelles would depends on the drug/polymer weight ratio. The values of drug loading capacity (DLC) and drug loading efficiency (DLE) could be calculated according to equations (1) and (2). The resulting DLC and DLE values with various drug/polymer weight ratios such as 1/1 and 0.5/1 were listed in Table 2. The different DLC and DLE values could be observed in NM1 and NM2 with various KGN molecule/polymer weight ratios such as 1/1 and 0.5/1, respectively. Relative high DLC and DLE values would be observed in the system of KGN and NM1 having a relative high drug/polymer weight ratio of 1/1. DLC and DLE values would be decreased in the system of KGN and NM2 having a relative low drug/polymer weight ratio of 0.5/1 as listed in Table 2.

**TABLE 2:** DLC and DLE of mPEG-Hz-*b*-PCL nanomicelles.

Sample	Drug/Polymer weight ratio	DLC% (%)	DLE% (%)
NM-1^a^	1/1	46.1	87.3
NM-2^b^	0.5/1	27.5	76.9

a NM-1:The mPEG-Hz-b-PCL, as a drug carrier with a relative high amount of KGN.

b NM-2:The mPEG-Hz-b-PCL, as a drug carrier with a relative low amount of KGN.

### *In vitro* Drug Release of KGN Molecule From KGN-Loaded mPEG-Hz-*b*-PCL Nanomicelles

**FIGURE 4:**
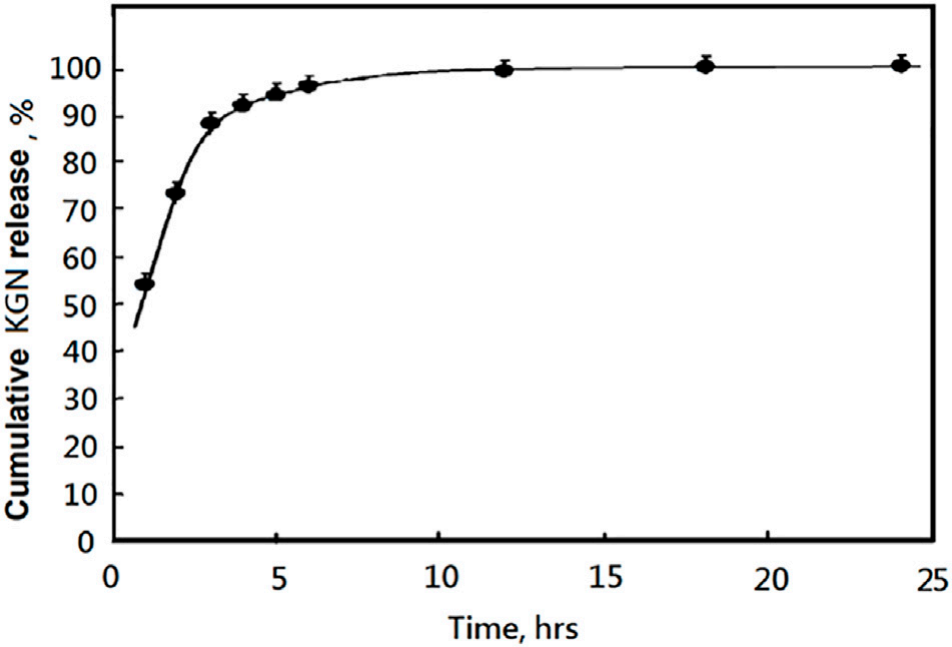
The released KGN percentages of KGN-loaded mPEG-Hz-*b*-PCL nananomicelles determined by HPLC under pH 7.4 at 37°C.

Figure 4 illustrates the *in vitro* drug release profiles of KGN-loaded mPEG-Hz-*b*-PCL nanomicelles in PBS buffer (pH 7.4) for KGN molecule. The release of KGN from the KGN-loaded mPEG-Hz-*b*-PCL nanomicelles showed an initial burst of ca.52.7% at the 1st hr and pH of 7.4. The cumulative release of KGN molecule persistently would increase with the following time as shown in Figure 4. After 24 h incubation, the cumulative release of KGN molecule was ca.99.9% for KGN-loaded mPEG-Hz-*b*-PCL nanomicelles. It could be concluded that KGN-loaded mPEG-Hz-*b*-PCL nanomicelles exhibited a remarkably fast drug release behavior of KGN molecule. This is probably because of the hydrophilic part of the KGN-loaded mPEG-Hz-***b***-PCL nanomicelles, which promotes the permeation of PBS buffer solution into the core of nanomicelles to facilitate KGN molecule release. Interestingly, the release behavior of KGN from the KGN-loaded mPEG-Hz-*b*-PCL nanomicelles under pH of 5.0 was quite different from that of KGN-loaded mPEG-Hz-*b*-PCL nanomicelles under pH of 7.4. Disintegration of KGN-loaded mPEG-Hz-*b*-PCL nanomicelles would be happen under acidic environments such as pH of 5.0 which were contributed to the bond breaking of hydrazone-linkage in mPEG-Hz-*b*-PCL, which would be hydrolyzed to hydrophobic PCL segments and hydrophilic mPEG segments.

**SCHEME 3:**
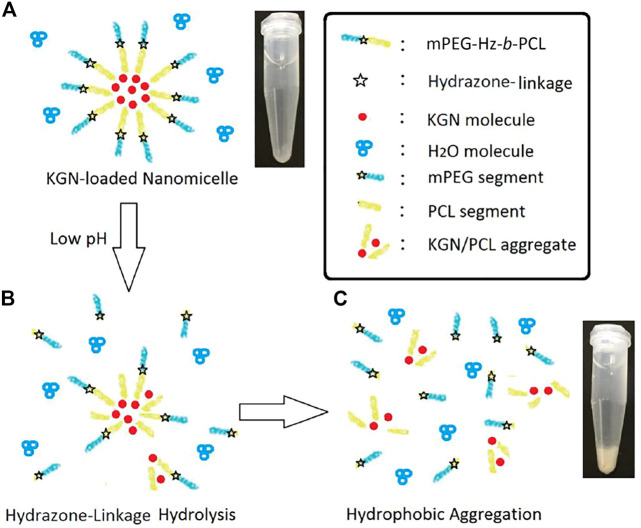
Proposed model of KGN-loaded mPEG-Hz-*b*-PCL micelles included a specific hydrazone-linkage structure which could respond to changes in the pH value of the surrounding environment in medical applications. (A)Formation of KGN-loaded nanomicelle, (B) hydrazone-linkage hydrolysis in low pH of the surrounding environment, and (C)hydrophobic aggregation in low pH of the surrounding environment.

About *in vitro* release of KGN molecule study, hydrolysis of KGN-loaded mPEG-Hz-*b*-PCL would not be happened at pH 7.4. Most of KGN molecules would be buried and kept in the core of mPEG-Hz-*b*-PCL nanomicelles (Scheme 3A). As the time increasing, the amount of KGN molecules would be released in the release medium. The amount of KGN molecule released from the KGN-loaded nanomicelle was calculated at specific time intervals as shown in Figure 4. However, hydrolysis of KGN-loaded mPEG-Hz-*b*-PCL would be rapidly happened in the acidic environment such as pH 5.0 (Scheme 3B). Because of the disintegration of KGN-loaded mPEG-Hz-*b*-PCL, most of KGN molecules would associate with bond-breaking PCL-containing segments and precipitates of associated KGN molecules/PCL-containing segments would be observed in the bottom as shown in Scheme 3C. The associated KGN molecules/PCL-containing segments sediment was separated from the release medium. Centrifugation was employed to separate the residual KGN-loaded mPEG-Hz-b-PCL nanomicelles from released dissolved KGN in the release medium. Although the residual unassociated KGN molecules could be slightly released in the release medium, residual unassociated KGN molecules was only observed <1.0% even over 24 h.

In this study, more than 90% of the KGN were released before 5 h at pH 7.4. This kind of release property is difficult to achieve the sustained release of KGN. The KGN molecules might be quickly released and lost in the physiological environment before being endocytosed by chondrocytes. Hence, this kind of micelle was difficult to be applied directly as a drug carrier for OA and a new deigned delivery system must be established. Also, *in vitro* MSCs chondrogenesis of KGN-loaded micelles might be designed depending on the resulting special drug release behaviors and designed drug delivery system for treatment of osteoarthritis (Zhu et al., 2017).

**FIGURE 5:**
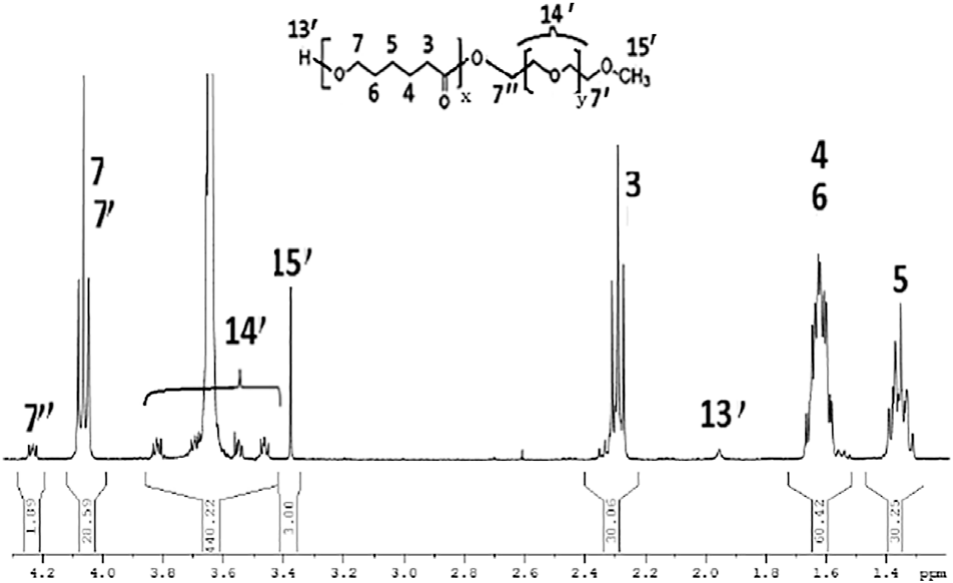
^1^H NMR spectrum of the model compound mPEG-*b*-PCL.

Furthermore, the model compound mPEG-*b*-PCL was prepared for identifying the acidic hydrolysis behavior of mPEG-*b*-PCL block copolymer’s main chain. ^1^H NMR spectrum of the synthesized mPEG-*b*-PCL block copolymer was showed in Figure 5. The formation of mPEG-*b*-PCL block copolymer could be confirmed from the terminated proton(H13′) of hydroxy end group in PCL segments at 1.98 ppm and terminated proton(H15′) of methoxyl end group in mPEG segment at 3.36 ppm. The characteristic peaks at 1.36 ppm, 1.56–1.63, 2.29, and 4.04 ppm were assigned to the methylene protons of -OCH_2_CH_2_CH_2_CH_2_CH_2_(H5), O(O)CCH_2_CH_2_CH_2_CH_2_CH_2_O(H4,H6), -O(CO)CH_2_(H3), and -OCH_2_- (H7,H7′) in the PCL segment of mPEG-*b*-PCL block polymer. The characteristic peaks at 3.36 ppm, 3.44–3.79 ppm, 4.25 ppm were attributed to the signals of CH_3_O-(H15′), -CH_2_CHO- and -CH_2_CHO-(H14′), -(CO)OCH_2_- (H7″) in the mPEG segment of mPEG-Hz-*b*-PCL block copolymer. The number average molecular weight (Mn) of the mPEG-Hz-b-PCL block copolymer could be calculated from 1H NMR spectrum as shown in Figure 5. Also, the methoxy polyethylene glycol (mPEG) weight fraction of the mPEG-*b*-PCL block copolymer could be calculated from the results of ^1^H NMR spectrum. The area ratio of OCH_2_CH_2_CH_2_CH_2_CH_2_(H3,H4,H5,H6; H) of PCL segment/-(CO)OCH_2_- (H7″; 2H) linkage between PCL segment and mPEG segment was calculated to be a ratio of 8H/2H = (30.25 + 30.06+60.42)/(1.89) by calculation of area of the characteristic peak at 1.36, 1.56–1.63, 2.29 ppm (H5, H4 and H6, H3) and the characteristic peak at 4.21 ppm (H7″). The “x” value of copolymer had been determined to be ca.16. Further, the area ratio of -CH_2_CHO- and -CH_2_CHO-(H14′) and terminated proton of methoxyl end group(H15′) was calculated to be a ratio of 4yH/3H = (440.22)/(3.00) by calculation of area of the characteristic peak at 3.44–3.79ppm (H14′) and the characteristic peak at 3.35 ppm (H15′). The “y” value of copolymer had been determined to be ca.110. The resulting mPEG-*b*-PCL with averaged molecular weight of 6,612 could be calculated. The resulting mPEG-*b*-PCL solution with a concentration of 5.00 × 10^−2^ mg/ml was kept in an acidic environment under pH of 5.0 for 24 h. The mPEG-*b*-PCL was precipitated by centrifugation. The ^1^H NMR spectrum (CDCl_3_) of mPEG-*b*-PCL exhibited same structure even in an acidic environment. The hydrolysis of hydrophobic PCL segments was not carried out in the acidic environment under pH of 5.0. Some literatures also showed these behaviors (Sunoqrot et al., 2017; Zhang et al., 2019; Wang et al., 2020; Park et al., 2021).

## Conclusion

In this study, a designed Kartogenin delivery system of pH-responsive mPEG-Hz-*b*-PCL nanomicelles was successfully prepared for treatment of osteoarthritis. These nanomicelles were stable, monodispersed, and low toxic. However, more than 90% of the KGN were released before 5 h at pH 7.4. This kind of release property would be difficult to achieve the sustained release of KGN and applied directly as a drug carrier for OA. It is necessary to build up a new deigned delivery system containing mPEG-Hz-*b*-PCL which would provide a possible potential for treatment of osteoarthritis in the future.

## Data Availability

The original contributions presented in the study are included in the article, further inquiries can be directed to the corresponding author.
